# Long noncoding RNA genes: conservation of sequence and brain expression among diverse amniotes

**DOI:** 10.1186/gb-2010-11-7-r72

**Published:** 2010-07-12

**Authors:** Rebecca A Chodroff, Leo Goodstadt, Tamara M Sirey, Peter L Oliver, Kay E Davies, Eric D Green, Zoltán Molnár, Chris P Ponting

**Affiliations:** 1 Department of Physiology, Anatomy, and Genetics, Le Gros Clark Building South Parks Road, University of Oxford, Oxford OX1 3QX, UK; 2 Genome Technology Branch, National Human Genome Research Institute, National Institutes of Health, 50 South Drive, Building 50, Room 5222, Bethesda, MD 20892, USA; 3 MRC Functional Genomics Unit, Le Gros Clark Building, South Parks Road, University of Oxford, Oxford OX1 3QX, UK

## Abstract

**Background:**

Long considered to be the building block of life, it is now apparent that protein is only one of many functional products generated by the eukaryotic genome. Indeed, more of the human genome is transcribed into noncoding sequence than into protein-coding sequence. Nevertheless, whilst we have developed a deep understanding of the relationships between evolutionary constraint and function for protein-coding sequence, little is known about these relationships for non-coding transcribed sequence. This dearth of information is partially attributable to a lack of established non-protein-coding RNA (ncRNA) orthologs among birds and mammals within sequence and expression databases.

**Results:**

Here, we performed a multi-disciplinary study of four highly conserved and brain-expressed transcripts selected from a list of mouse long intergenic noncoding RNA (lncRNA) loci that generally show pronounced evolutionary constraint within their putative promoter regions and across exon-intron boundaries. We identify some of the first lncRNA orthologs present in birds (chicken), marsupial (opossum), and eutherian mammals (mouse), and investigate whether they exhibit conservation of brain expression. In contrast to conventional protein-coding genes, the sequences, transcriptional start sites, exon structures, and lengths for these non-coding genes are all highly variable.

**Conclusions:**

The biological relevance of lncRNAs would be highly questionable if they were limited to closely related phyla. Instead, their preservation across diverse amniotes, their apparent conservation in exon structure, and similarities in their pattern of brain expression during embryonic and early postnatal stages together indicate that these are functional RNA molecules, of which some have roles in vertebrate brain development.

## Background

Whilst only approximately 1.06% of the human genome appears to encode protein [1,2] at least four times this amount is transcribed into stable non-protein-coding RNA (ncRNA) transcripts [3-5]. Unfortunately, the biological relevance of the vast majority of this extensive and interleaving network of coding RNAs and ncRNAs remains far from clear. One possibility is that many ncRNAs result simply from transcriptional 'noise'. If so, their sequence and transcription might be expected not to be conserved outside of restricted phyletic lineages. Indeed, the finding that only 14% of the well-defined mouse long intergenic ncRNAs (lncRNAs) identified in the FANTOM projects [6,7] have a transcribed ortholog in human (based on analyses of known EST and cDNA data sets) [2] argues against their functionality. Similarly, known human intergenic lncRNA loci are generally not conserved in sequence at statistically significant levels in the mouse genome [3,8,9], and there is little evidence for conserved expression of intergenic regions (including lncRNAs) between mouse and human [10].

On the other hand, our preconceptions of lncRNA functionality might be greatly prejudiced by our long-standing knowledge of protein evolution. Just because functional protein-coding sequence is highly constrained, this need not necessarily imply that largely unconstrained non-protein-coding sequence, free from the need of maintaining an ORF and producing a thermodynamically stable protein product, is not functional. Indeed, even well-known examples of functional mammalian lncRNAs, such as *Gomafu *[11], *Evf-2 *[12], *XIST *[13], *Air *[14], and *HOTAIR *[9], exhibit poor sequence conservation across species. Moreover, there is evidence for significant, albeit modest, evolutionary constraint within lncRNA loci compared to neutrally evolving DNA [15-18]. In addition, as with mRNAs, many lncRNAs are subject to splicing, polyadenylation, and other post-transcriptional modifications, and their loci tend to be associated with particular chromatin marks [15]. However, whether the observed chromatin marks and purifying selection are most frequently directed towards the transcribed lncRNA, the process of transcription, or the underlying DNA sequence remains unknown [19-21].

In support of functional roles for lncRNA loci, many lncRNAs have been shown to be developmentally regulated and/or expressed in specific tissues. For example, a computational analysis of *in situ *hybridization data from the Allen Brain Atlas identified 849 lncRNAs (out of 1,328 examined) showing specific expression patterns in adult mouse brain [22]. Similarly, 945 lncRNAs were found to be expressed above background levels in a microarray screen of mouse embryonic stem cells at various stages of differentiation [23]. A follow-up study found that 5% of approximately 3,600 analyzed lncRNAs are differentially expressed in forebrain-derived mouse neural stem cells subjected to various developmental paradigms [24]. Such regulated expression patterns can perhaps be attributed to lncRNA loci tending to cluster near brain-expressed protein-coding genes and transcription factor-encoding genes associated with development [15,17,25].

Nevertheless, it is important to stress that the above-mentioned studies focused on only one species, namely the laboratory mouse. There is a clear and substantial need to investigate the evolution and expression of specific lncRNA loci for more diverse species, for example birds, whose lineage separated from that of mammals approximately 310 million years ago [26]. However, few, if any, studies have identified orthologous lncRNAs shared between birds and mammals, let alone investigated either their expression in homologous developmental fields or adult anatomical structures, or their molecular functions. Whilst one study found that *Sox2ot *is both dynamically regulated and transcribed from highly conserved elements in chicken and zebrafish [27], this locus overlaps with a protein-coding gene (*Sox2*), a pluripotency regulator, and thus is not intergenic. A more comprehensive study of full-length chicken cDNA sequences identified 30 transcripts that could be aligned with RIKEN-identified mouse lncRNAs, although their expression in developing chick embryos was undetectable [28]. Even *Xist*, which is involved in chromosome-wide × inactivation in eutherians, is not conserved as a lncRNA in birds, as its avian ortholog is protein-coding [29].

In this study, we used a multi-disciplinary approach to investigate a select group of highly conserved lncRNAs that are expressed within the embryonic and early postnatal mouse brain. We report the characterization of four such lncRNAs, demonstrating that they are expressed at experimentally detectable levels, are tissue-specific and developmentally regulated, and are conserved in transcript structure and expression pattern across diverse amniotes during brain development. To our knowledge, this is the first description and investigation of lncRNA loci with orthologs present in eutheria, metatheria (marsupials), and birds. As these lncRNAs do not differ substantially from protein-coding genes in their sequence or expression properties, we propose that they are novel RNA genes that are likely to confer important functions among these diverse amniotes. Our observations provide the first indications that investigation of lncRNA orthologs in amniote model organisms will be informative about their contributions to human biology.

## Results

### lncRNA selection

**Figure 1:**
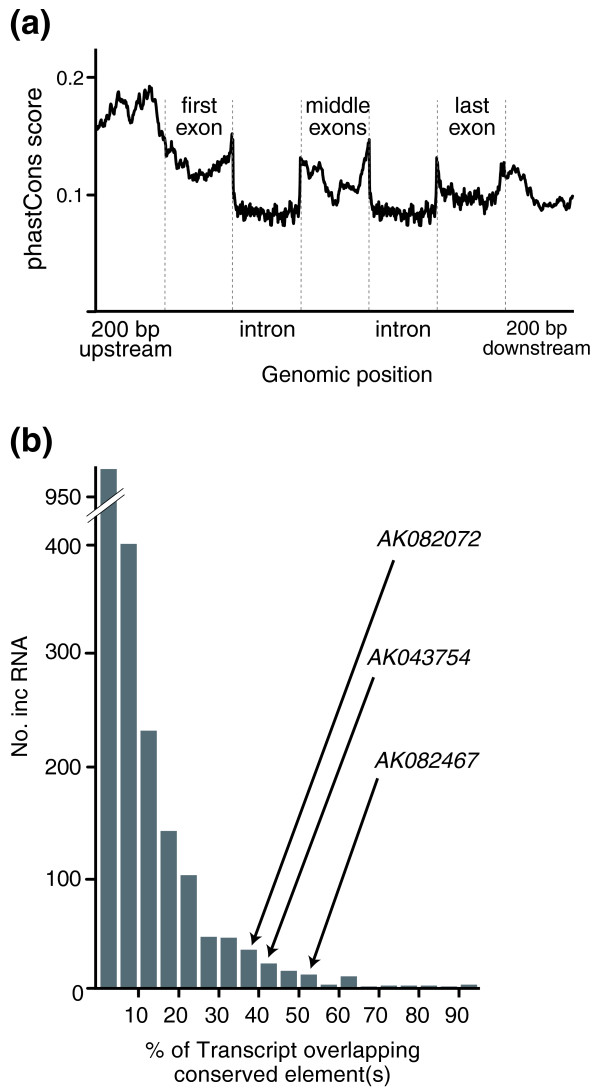
**Sequence conservation among lncRNAs**. **(a) **Conservation across a generic lncRNA locus, based on 877 mouse multi-exon lncRNAs. We sampled 200 evenly spaced bases across each region listed, with regions containing fewer than 200 bases sampled entirely. The graph shows the average vertebrate phastCons score at each genomic position across all multi-exon lncRNA loci. Note phastCons score peaks within the putative promoter region (200 bp upstream) and near donor and acceptor splice sites (analysis inspired by Figure 25a in [31]). (**b**) Overlap between vertebrate phastCons-predicted conserved elements and mouse lncRNA exons. Of 2,055 lncRNAs with signatures of purifying selection initially identified in mouse [18], 1,095 contain exons that overlap phastCons-predicted vertebrate conserved elements (log-odds score range 1 to 1,000) [30]. Depicted is a histogram showing the percentage of each lncRNA transcript that overlaps a phastCons-predicted vertebrate conserved element. The relative positions of three selected lncRNAs (*AK082072*, *AK043754*, and *AK082467 *with overlaps of 36.7, 44.8, and 51.7%, respectively) are shown.

We started with a set of 3,122 well-characterized intergenic lncRNAs derived from FANTOM 2 and 3 consortia collections of full-length noncoding transcripts in the mouse [6,7,18]. While transcripts with evidence of protein-coding capacity had already been discarded, we removed additional lncRNAs that overlap either with more-recently annotated mouse protein-coding genes or with alignable protein-coding genes from other species. We also discarded lncRNAs transcribed in close proximity (<5 kb) of annotated protein-coding genes in order to reduce the chances of inadvertently considering untranslated regions or alternative transcripts of these genes. Of the remaining set of 2,055 lncRNA transcripts, 1,209 (59%) harbor strongly constrained sequence, based on overlap with phastCons-predicted conserved elements (Figure 1b) [30], consistent with a recent report [16]. On average, 10.6% and 10.9% of the lncRNA sequences (including and excluding introns, respectively) overlap phastCons-predicted conserved elements.

To compare the evolution of lncRNA loci with protein-coding gene evolution, we next constructed a generic locus from 877 multi-exon lncRNA loci, and annotated it according to the presence of conserved sequence elements (Figure 1a). A similar portrait of evolutionary conservation for protein-coding genes was presented by the Mouse Genome Sequencing Consortium (Figure 25a in [31]). As seen for protein-coding genes, sequence conservation is not uniformly distributed across various features (exons, introns, and upstream and downstream regions) of a generic multi-exon lncRNA locus (Figure 1a). The putative core promoter region (here defined as 200 bp upstream of each lncRNA transcription start site (TSS)) is generally under greater evolutionary constraint than lncRNA exonic sequence, in agreement with previous reports [6,16,18]. Constraint peaks at 0.19 (range between 0 and 1), 43 bp upstream of the normalized TSS, as previously observed for human and mouse promoter sequence [32]. Just as for protein-coding genes [31], the generic lncRNA locus' first, middle and last exons tend to be under greater evolutionary constraint than its introns, with average phastCons scores peaking in close proximity to splice sites.

To establish whether lncRNAs are conserved in expression as well as in sequence, we sought to select a small number of mouse lncRNAs and investigate their putative orthologs in other amniotes, namely the marsupial opossum (*Monodelphis domestica*) and the chicken (*Gallus gallus*). We chose lncRNAs that are highly conserved, developmentally regulated, and brain-expressed. These criteria were used because our previous study [17] found that constrained lncRNAs with significantly suppressed human-mouse nucleotide substitution rates tended to be expressed in the mouse brain and, when developmentally expressed, to be transcribed near protein-coding genes involved in transcriptional regulation.

Accordingly, we selected three lncRNAs, each having extensive overlap with phastCons-predicted conserved elements (Figure 1b) and each expressed in embryonic or neonatal brain based on the origin of the cDNA library from which they were identified. Here, we refer to these three lncRNAs and their genomic loci according to their database accession numbers: *AK082072*, *AK082467*, and *AK043754*.

### Structure of selected lncRNA loci

**Figure 2:**
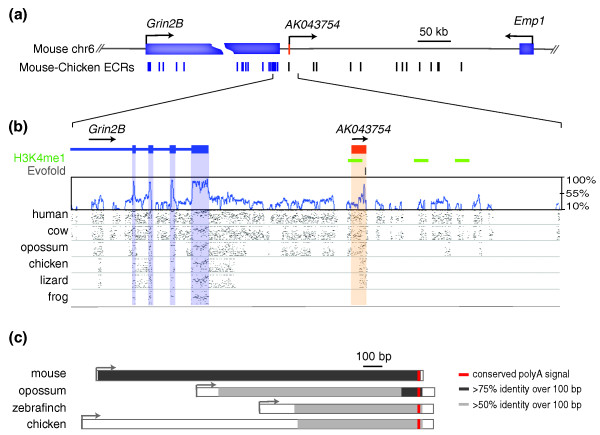
**Evolutionary constraint of *AK043754***. **(a) **The genomic region of mouse chromosome 6 (chr6) encompassing the lncRNA locus *AK043754 *(1.7 kb) is depicted. Note the locations of flanking protein-coding genes: *Grin2B *(glutamate receptor, ionotropic, NMDA2B (N-methyl-D-aspartic acid)) and *Emp1 *(epithelial membrane protein 1). Also shown are the positions of mouse-chicken ECRs (evolutionarily conserved regions at least 100 bp in size with 70% sequence identity between the mouse and chicken genomes); ECRs within protein-coding regions are shown in blue. **(b) **A more detailed representation of *AK043754 *(single exon highlighted in orange) and its immediate flanking regions, including the 3' end of *Grin2B*. Below the gene structures are the positions of H3K4me1 chromatin marks (green) detected in mouse embryonic stem cells (obtained from UCSC Genome Browser), EvoFold predictions of RNA secondary structures (grey), a SinicView conservation plot [68] based on a 21-vertebrate multispecies sequence alignment (using Threaded Blockset Aligner) generated with mouse as the reference sequence, and Gmaj [66] views of alignments between mouse and the indicated species' sequences (note the detected homology with the orthologous lizard and chicken, but not frog, sequences). **(c) **Conservation and relative sizes of *AK043754 *orthologs in various species. The TSSs (arrows) and transcript lengths are depicted in each case. Note the conserved position of a polyA signal (red) and increased sequence conservation (relative to the mouse sequence) towards the 3' end. ECR, evolutionarily conserved region.

**Figure 3:**
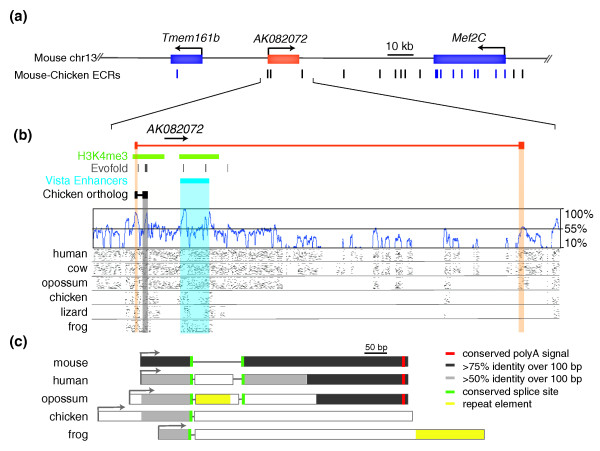
**Evolutionary constraint of *AK082072***. **(a) **The genomic region of mouse chromosome 13 (chr13) encompassing lncRNA *AK082072 *(523 bp) is depicted. Note the locations of the flanking protein-coding genes: *Tmem161b *(transmembrane protein 161b) and *Mef2C *(myocyte enhancer factor 2C). **(b) **A more detailed representation of *AK082072 *(exons highlighted in orange) and its immediate flanking regions. Below the gene structures are the positions of H3K4me3 chromatin marks (green) detected in mouse brain, VISTA conserved non-coding midbrain enhancer element 268 (obtained from the UCSC Genome Browser), and a BLAT alignment of the chicken *AK082072 *ortholog, as well as similar tracks as those in Figure 2b. Note the detected homology with orthologous frog sequence in exon 1. **(c) **Conservation and relative sizes of *AK082072 *orthologs in various species. Note the sequence conservation (relative to the mouse sequence) at both the 5' and 3' ends and the conserved position of splice sites (green). Unlike the other vertebrate genomes considered, the zebra finch genome did not align to the proximal promoter or first exon of mouse *AK082072*. This apparent lack of sequence identity might reflect either an unannotated gap in its genome assembly or rapidly evolving sequence within its orthologous genomic region. Other details are provided in the legend to Figure 2. ECR, evolutionarily conserved region.

**Figure 4:**
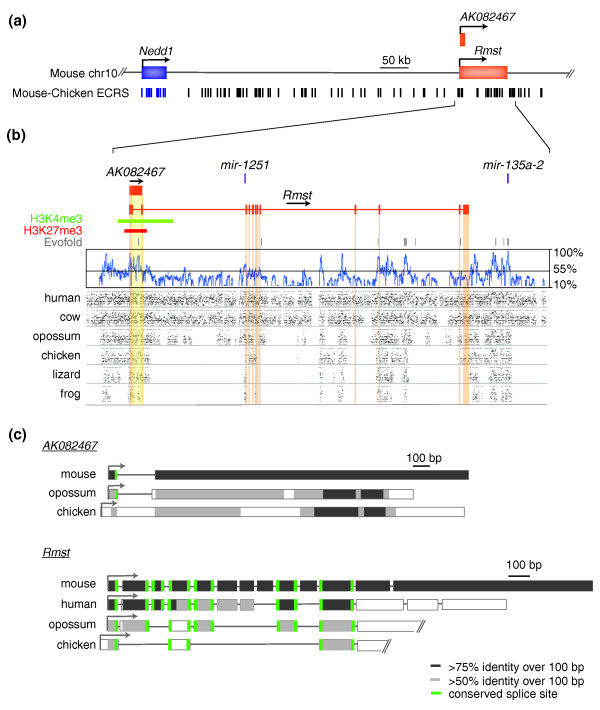
**Evolutionary constraint of *AK082467 *and *Rmst***. **(a) **The genomic region of mouse chromosome 10 (chr 10) encompassing lncRNAs *AK082467 *(2.7 kb) and *Rmst *(2.7 kb) is depicted. Note the presence of the protein-coding gene *Nedd1 *(neural precursor expressed developmentally down-regulated protein 1) upstream of *AK082467 *and *Rmst*. **(b) **A more detailed representation of *AK082467 *and *Rmst *(exons highlighted in yellow and orange, respectively), microRNAs *mir-1251 *and *mir-135a-2*, and their immediate flanking regions. Below the gene structures are the positions of H3K4me3 (green) and H3K27me3 (red) chromatin marks detected in mouse brain (obtained from the UCSC Genome Browser) as well as similar tracks as those in Figure 2b. Note the detected homology with orthologous frog sequence in *Rmst *exons 1, 2, 4, and 11. **(c) **Conservation and relative sizes of *AK082467 *and *Rmst *orthologs in various species. Note the conserved splice sites (green bars) in mouse *Rmst *exons 1, 4, and 11 as well as the sequence conservation (relative to mouse sequence) in exons 1 and 11, but differences in total exon number among species. The 3' ends of opossum and chicken orthologs have not been experimentally verified. Other details are provided in the legend to Figure 2. ECR, evolutionarily conserved region.

The three selected lncRNA loci harbor elements that are more usually associated with protein-coding genes. These include GT-AG donor-acceptor splice sites, polyadenylation signals, and chromatin marks in their putative promoter regions (Figures 2b,c, 3b,c and 4b,c; Figure S1 in Additional file 1). Aceview annotations [33] indicate an unspliced (single exon) transcript and single promoter for the *AK043754 *locus (spanning 1.75 kb on mouse chromosome 6qG1), a single canonical GT-AG intron and promoter for the *AK082072 *locus (39.7 kb on mouse chromosome 13qC3), and 31 different GT-AG introns in at least 16 different mRNA splice variants and 6 probable alternative promoters for the *AK082467 *locus (94 kb on mouse chromosome 10qC2). Each lncRNA sequence is supported by several GenBank cDNA records, representing cDNAs derived primarily from mouse embryonic or neonatal central nervous system tissues, including hypothalamus, diencephalon, cortex, cerebellum, and spinal cord. Many of the supporting GenBank records additionally support poly(A) and 5' cap structures, indicating that each lncRNA is most likely transcribed by RNA polymerase II. Chromatin marks from either mouse embryonic stem cells or adult mouse whole brain [34] are present at each putative lncRNA promoter (Figures 2b, 3b and 4b).

In contrast to most protein-coding genes, the lncRNA loci each harbor at least one Evofold-predicted RNA secondary structure (Figures 2b, 3b and 4b) [35]. This reflects the general tendency of conserved brain-expressed lncRNA loci to contain such structures [17]. The three lncRNA transcripts each lack long (>100 amino acids) ORFs. While it remains possible that the lncRNAs encode short peptides, there is no evidence for constraint on their protein-coding capacity, as the frequencies of synonymous and non-synonymous substitutions across eutherians are roughly equal (that is, dN/dS ≈ 1 ± 0.16) for the longest predicted ORF of each lncRNA [36].

These findings imply that the three selected transcripts might be functional noncoding RNA genes. *AK082467 *is an alternative splice variant that contains the first three exons and retains the second intron of a previously described long noncoding RNA, *Rmst *(rhabdomyosarcoma 2 associated transcript, also known as *NCRMS*); the human *RMST *ortholog was initially identified as a differentially expressed transcript in alveolar versus embryonic rhabdomyosarcoma (a malignant soft tumor tissue), but its function remains undocumented [37]. To our knowledge, *AK043754 *and *AK082072 *have not been experimentally investigated. To examine their potential functions, we first studied the expression patterns of the three lncRNAs during mouse development.

### Expression of selected lncRNAs in mouse

Analysis of the three selected lncRNAs by *in situ *hybridization of mouse tissues at different developmental time points revealed that each exhibits a specific expression pattern that, in general, is restricted to the brain. Our findings further suggest their expression is tightly regulated, as opposed to stochastic background transcription.

**Figure 5:**
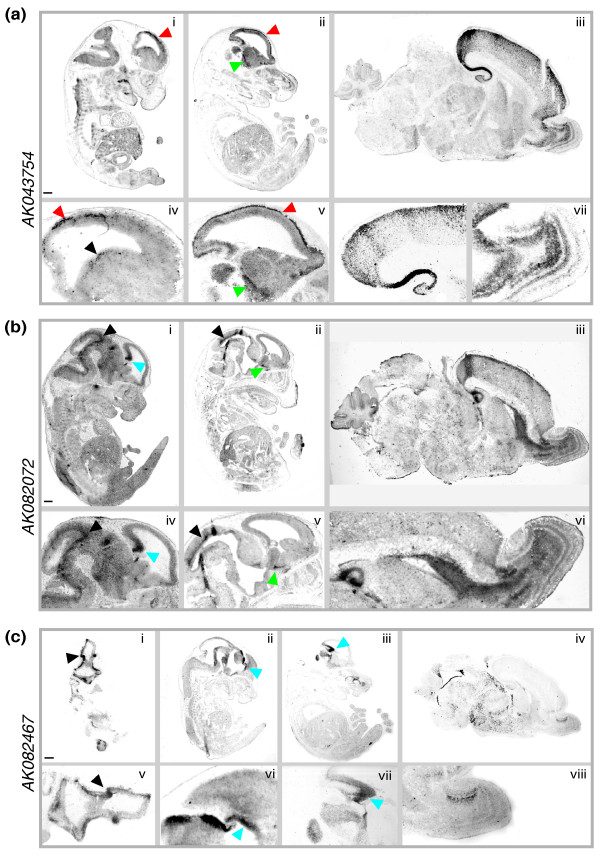
**lncRNAs are specifically expressed and developmentally regulated in the mouse brain**. **(a-c) **Digoxigenin-labeled riboprobes complementary to *AK043754 *(a), *AK082072 *(b), and *AK082467 *(c) were hybridized to sagittal sections of C57BL/6J mouse brains at different development stages (E9, E13, E17, and P3). (a) The *AK043754 *probe hybridized to the first generated cell layer of the preplate or primordial plexiform zone (red arrowheads) at E13 (i, iv) and E17 (ii, v), the ventricular zone of the medial and lateral ganglionic eminences (black arrowhead) at E13, the latero-caudal migratory path from the basal telencephalon to the striatum (green arrowhead) at E17 (ii, v), and the hippocampus (iii, vi) and the olfactory bulb (iii, vii) at P3. Scale bar (shown in (i)) is 500 μm in (i), 543 μm in (ii), 322 μm in (iii), 292 μm in (iv), 300 μm in (v), 167 μm in (vi), and 214 μm in (vii). (b) The *AK082072 *probe hybridized to the hem of the embryonic cerebral cortex (blue arrowheads) and the roof of the midbrain (black arrowheads) at E13 (i, iv) and E17 (ii, v), and to the hippocampus (iii, vi), rostral migratory stream (iii, vi), and internal plexiform and granule cell layer of the olfactory bulb (iii, vi) at P3. Scale bar (shown in (i)) is 500 μm in (i), 595 μm in (ii), 422 μm in (iii), 357 μm in (iv), 386 μm in (v), and 311 μm in (vi). (c) The *AK082467 *probe hybridized to the optic stalk (black arrowheads) at E9 (i, v), the cortical hem (blue arrowheads) at E13 (ii, vi) and E17 (ii, vii), and the accessory olfactory bulb (iii, viii) at P3. Scale bar (shown in i)) is 500 μm in (i), 637 μm in (ii), 684 μm in (iii), 522 μm in (iv), 182 μm in (v), 177 μm in (vi), 176 μm in (vii), and 110 μm in (viii).

*AK043754 *is initially expressed in the primordial plexiform layer or preplate. This is the first of the developmental cell layers to appear during mammalian embryogenesis and is, most likely, homologous to the simpler amphibian and avian cortical structures (Figure 5a(i,ii,iv,v)) [38]. At embryonic day 17 (E17), *AK043754 *is expressed prominently within the marginal zone along the pial surface in a pattern similar to that of reelin-expressing Cajal-Retzius cells. Of note, the expressed transcript is also present within the ventricular zone of the ganglionic eminence, a source of GABAergic migratory neurons (including some Cajal-Retzius cells) that ultimately colonize the marginal zone, intermediate zone, and subplate; this suggests that *AK043754*-expressing cells might originate in the ganglionic eminence and then migrate to the preplate and marginal zone [39]. Reinforcing this transcript's potential association with inhibitory GABAergic neurons, hybridization is also seen in the latero-caudal migratory path of interneurons from the basal telencephalon to the striatum. This is best illustrated at stage E17 and within the internal granule cell layers of the olfactory bulb at postnatal day 3 (P3; Figure 5a(vii)).

Cells expressing *AK082072 *at stage E13 primarily populate the roof of the midbrain and the cortical hem (the most caudomedial edge of the telencephalic neuroepithelium), one of the major patterning centers of the developing telencephalon and, as recently shown by Monuki and Tole and colleagues, a hippocampal precursor (Figure 5b(i,iv)) [40,41]. By stage E17, expression continues to be apparent within the roof of the midbrain, and, as illustrated at higher magnification, is strongest in the soma and outward projections of cells lining the midbrain ventricle (Figure 5b(v)). Also visible in the E17 image is the expression of *AK082072 *along the caudal ganglionic eminence, a major source of GABAergic neurons that preferentially migrate caudally to the caudal cortex and hippocampus [42]. At postnatal stages, *AK082072 *expression is restricted to the hippocampus (mostly within CA1), the rostral migratory stream, and the internal plexiform and granule cell layer of the olfactory bulb. Reinforcing our observations, a previous independent study that utilized a probe designed from another region of the *AK082072 *transcript yielded similar results [43].

*AK082467 *is expressed early in mouse brain development, with its transcription mostly attenuated after birth. The antisense riboprobe designed to an intron-spanning region of this lncRNA transcript partially overlaps the 5' region of *Rmst*, such that all observations could reflect the expression pattern(s) of one or both of these transcripts. Consistent with the expression pattern of *Rmst *described by Bouchard *et al. *[44], our riboprobe hybridized to the mid-hindbrain organizer region in developing mouse embryos, most clearly illustrated in Figure 5c(ii). We also found expression in two additional *Pax2-*expressing regions, including the optic stalk at stage E9 and within the accessory olfactory bulb postnatally (Figure 5c(i,iv)).

### lncRNA orthologs in other vertebrates

*AK082072*, *AK082467*, *Rmst*, and *AK043754 *are each transcribed from regions of the mouse genome whose sequence aligns to vertebrate genome sequences from species at least as distantly related as chicken, with greater than 80% nucleotide identity within some intervals. We sought to determine whether conservation in lncRNA sequence also extends to conservation in the expression of these lncRNAs among diverse vertebrate species. In order to identify orthologs in other vertebrates, we aligned genomic sequences orthologous to each lncRNA locus from species ranging from frog to human, and including birds and marsupials (see Materials and methods; Figures 2b, 3b and 4b).

Each lncRNA locus and its closest flanking protein-coding genes show conserved synteny across amniotic species from mouse to chicken, and a portion of each mouse lncRNA locus aligns to all the genomic sequences we analyzed (Figures 2a, 3a and 4a). The patterns of nucleotide conservation for these lncRNA loci exemplify the more general trends we observed for all such loci, including greater conservation near exon boundaries (Figure 1a). In these respects, these lncRNA loci differ markedly from protein-coding genes, which typically contain more uniformly distributed and strong conservation within exons [31].

#### AK043754

Blocks of aligned sequence with at least 70% nucleotide identity across all the examined amniote species are restricted to the 3' end (approximately 500 bp) of *AK043754 *(Figure 2). We could find no evidence of *AK043754*-aligning sequence within non-amniote vertebrate genomes, suggesting that this locus has either evolved extremely rapidly or originated within the amniote lineage after divergence from other vertebrates. The sequence of the putative proximal promoter, presumed to reside within the 400 bp upstream of the TSS, aligns to orthologous sequences in metatheria and eutheria; such orthologous sequence could not be identified in monotremata (platypus) and non-mammalian vertebrates. Finally, a polyadenylation signal (ATAAA) located 30 bp upstream of the 3' end of *AK043754 *in mouse is present in all examined amniote sequences.

Guided by the multi-species sequence alignments, we cloned the *AK043754 *orthologs from opossum and chicken poly(A)-selected reverse-transcribed cDNA. As illustrated in Figure 2c, the orthologous opossum and chicken sequences (as well as the orthologous zebra finch sequence [GenBank: DQ213170]) align to the mouse *AK043754 *sequence. Based on BlastN local alignments, the opossum (1,307 bp), chicken (1,912 bp), and zebra finch (938 bp) transcripts share approximately 38%, 29%, and 29% nucleotide sequence identity with the mouse transcript, respectively. Consistent with the multi-species genome sequence alignment, each transcript has a unique (non-aligning) TSS (indicated by grey arrows), but harbors a conserved poly(A) signal (red band) and 3' end. As with mouse *AK043754*, the examined orthologs lack long or conserved ORFs, indicating that this locus is unlikely to have possessed protein-coding capacity over the span of amniote evolution.

#### AK082072

Orthologous sequences in each of the 16 vertebrate genomes we examined (with one exception - see below) aligned to the proximal promoter and first exon of mouse *AK082072 *with sequence identities exceeding 85% (Figure 3b). Notably, a 5' consensus splice-site sequence (MAG|GTRAG) for U2 introns in pre-mRNA is constrained. However, sequence conservation of the second exon, including an adjacent 3' AG acceptor site and poly(A) signal, is detectable only in mammals, suggesting that this region might have arisen within the mammalian lineage after divergence from other amniotes.

*AK082072 *orthologs were identified in frog (754 bp), chicken (759 bp), and human (553 bp) ([GenBank: CX847574.1, CR35248.1, DA317999.1], respectively) from a BLASTn query of the NCBI (nr/nt) database. In addition, we cloned and sequenced the full-length (725 bp) opossum ortholog from poly(A)-selected reverse-transcribed cDNA. Based on the resulting BLASTn alignments, we found that the frog, chicken, opossum, and human sequences share approximately 11%, 21%, 53%, and 67% sequence identity, respectively, with their mouse ortholog (Figure 3c). Consistent with the multi-species genome sequence alignment, all transcripts utilize a conserved 5' donor site. By contrast, only the mammalian transcripts use the predicted 3' acceptor site and terminate immediately after the predicted poly(A) signal (depicted as blue and red bands, respectively, in Figure 3c).

While the relative structure of the first and last exons is conserved across therian mammals, the opossum and human orthologs contain an additional and non-homologous central exon, in each case buttressed by non-conserved AG/GT acceptor/donor sites and residing within poorly constrained genomic sequence. In fact, the opossum middle exon lies within a genomic region containing a MAR1 element (a tRNA-derived SINE (short interspersed element) specific to *M. domestica *[45]).

The terminal mammalian *AK082072 *exons lack demonstrable homology with those in the chicken and frog orthologs (Figure 3b). The second exon in chicken *AK082072 *is transcribed from an evolutionarily conserved region that shares >70% sequence identity with the orthologous mouse sequence (highlighted in grey) across 200 bp and harbors a poly(A) signal with 100% sequence conservation in all examined vertebrates except zebra finch. While suggestive of a highly conserved exon, we were unable to clone similar splice variants from either mouse or opossum cDNA. In contrast, the second exon of frog *AK082072 *appears to be specific to amphibians and, like opossum *AK082072*, includes a repeat element, in this case a *X. tropicalis *DNA transposon hAT.

#### AK082467/Rmst

*AK082467 *and *Rmst *orthologs from human to frog also exhibit >70% sequence identity over their proximal promoters, first exons, and 5' splice donor sites (Figure 4b). In all examined eutherians, we identified putative two-exon *AK082467 *orthologs that share a TSS, splice site, and exonic structure. While genomic regions containing the second exon of *AK082467 *share at least 60% sequence identity among the examined vertebrates, the non-eutherian vertebrates lack an upstream 3' acceptor site; hence, we expected either unspliced or differentially spliced orthologs in these species. Indeed, we cloned unspliced and differentially spliced *AK082467 *orthologs from chicken (30% sequence identity) and opossum (26% sequence identity) cDNA, respectively, each sharing similar 5' and 3' ends with mouse *AK082467 *(Figure 4c). The opossum *AK082467 *3' acceptor site is not conserved, as it aligns approximately 10 bp upstream of that in mouse, although this may reflect inaccuracies in the sequence alignment. Chicken *AK082467 *contains an additional approximately 200-bp stretch that spans the mouse intronic region. Importantly, the identified mammalian intron in *AK082467 *(approximately 320 bp), which is almost entirely composed of simple repeats, is not alignable to chicken or to other non-mammalian vertebrate genomes. Also, we were unable to identify a poly(A) signal within the *AK082467 *orthologs despite the fact that the transcripts were derived from poly(A)-selected cDNA, suggesting that the isolated transcripts were either unpolyadenylated contaminants within our cDNA samples or that the transcripts are recapped derivatives of larger RNA molecules.

Our multi-species sequence alignment (Figure 4b) revealed that only exons 1, 4, and 11 of mouse *Rmst *share the same exonic structure (including alignable donor and acceptor splice sites) across the examined vertebrates. At least one >50-bp stretch of >60% sequence identity resides within each of these exons. Sequences of the remaining mouse exons align to regions of varying sequence conservation among mammals, suggesting relaxed evolutionary constraint on their structures. Accordingly, we predicted vertebrate *Rmst *orthologs containing at least three conserved exons and a variable number of total exons. Of note, we also identified a eutherian-specific poly(A) signal residing approximately 25 bp upstream of the termination site within the mouse transcript, suggesting that other eutherians also share the same transcription stop site.

We cloned and sequenced the chicken and opossum *Rmst *orthologs, which contain four and seven exons, respectively. While we only identified one splice variant for each species, alternative transcripts could exist. Alignment of the identified orthologs along with the mouse and human [GenBank: NR_024037] *Rmst *sequences revealed striking conservation of the structures of exons 1, 4, and 11 and of the sequences of exons 1 and 11 (Figure 4c). In contrast, the mouse, opossum, and chicken *Rmst *exon 4 orthologs share <50% sequence identity. Furthermore, the overall sequence identity, calculated by BLASTn, between mouse *Rmst *and the chicken, opossum, and human orthologs is only 4%, 7%, and 22%, respectively.

### Expression of selected lncRNA orthologs in the developing brain

Given the evidence that lncRNA orthologs are transcribed in diverse species, we next sought to determine whether the tissue pattern of transcription is similarly conserved. Indeed, we identified numerous homologous ESTs and cDNAs from nervous system tissue isolated from diverse species (human to zebra finch; Table 1).

**Table 1:** *AK043754, AK082072*, and *AK082467 *orthologs among vertebrates

lncRNA	Species (common name)	GenBank accession	Tissue type	Dev. stage
*AK043754*	*M. musculus *(mouse)	[Genbank:AK043754]*	Cortex	Neonate
	*R. norvegicus *(rat)	[Genbank:BF565173]	Brain	Adult
	*C. jacchus *(marmoset)	[Genbank:EH380404]	Hippocampus	Adult
	*H. sapiens *(human)	[Genbank:DB326634]	Brain	Fetal
	*B. taurus *(cow)	[Genbank:CO886535]	Brain	Adult
	*S. scrofa *(pig)	[Genbank:EW186118]	Cerebellum	Fetal
	*T. guttata *(zebra finch)	[Genbank:DV959637]	Brain	Pooled
				
*AK082072*	*M. musculus *(mouse)	[Genbank:AK082072]*	Cerebellum	Neonate
	*R. norvegicus *(rat)	[Genbank:CB798977]	Hypothalamus	Unknown
	*M. fascicularis *(macaque)	[Genbank:CJ466564]	Parietal lobe	Adult
	*H. sapiens *(human)	[Genbank:DA317999]	Hippocampus	Unknown
	*C. lupus familiaris *(dog)	[Genbank:CO685831]	Kidney	Adult
	*B. taurus *(cattle)	[Genbank:DV836210]	Hypothalamus	Adult
	*S. scrofa *(pig)	[Genbank:EV900652]	Cerebellum	Unknown
	*G. gallus *(chicken)	[Genbank:BU232759]	Head	Embryo
				
*AK082467/Rmst*	*M. musculus *(mouse)	[Genbank:AK082467]*	Cerebellum	Neonate
	*M. musculus *(mouse)	[Genbank:AK086758]*	Head	Embryo
	*R. norvegicus *(rat)	[Genbank:BF397583]	Whole embryo	Embryo
	*H. sapiens *(human)	[Genbank:DA347802]	Substantia nigra	Unknown
	*C. lupus familiaris *(dog)	[Genbank:CO586030]	Brain	Adult
	*B. taurus *(cow)	[Genbank:CB447323]	Pooled	Unknown
	*S. scrofa *(pig)	[Genbank:BI405055]	Anterior pituitary	Adult

*Sequences used as queries in BLASTN searches against the NCBI nr database to identify orthologous ESTs. The cut-off for significance was set at *E*-value < 1 ×10^-10^.

To observe lncRNA expression at a finer resolution, we performed *in situ *hybridization of mouse, opossum, and chicken brains harvested at early and late embryonic stages, using probes specific to approximately 300-bp portions of phastCons conserved elements within *AK043754*, *AK082072*, and *AK082467 *exons. While the expression patterns of the lncRNA orthologs are not identical among these species, we encountered evidence of spatio-temporal regulation for each locus, with transcription typically regionally restricted within embryonic and neonatal brain tissue. Many of these regions have been implicated in the evolution of the mammalian cerebral cortex [46,47].

**Figure 6:**
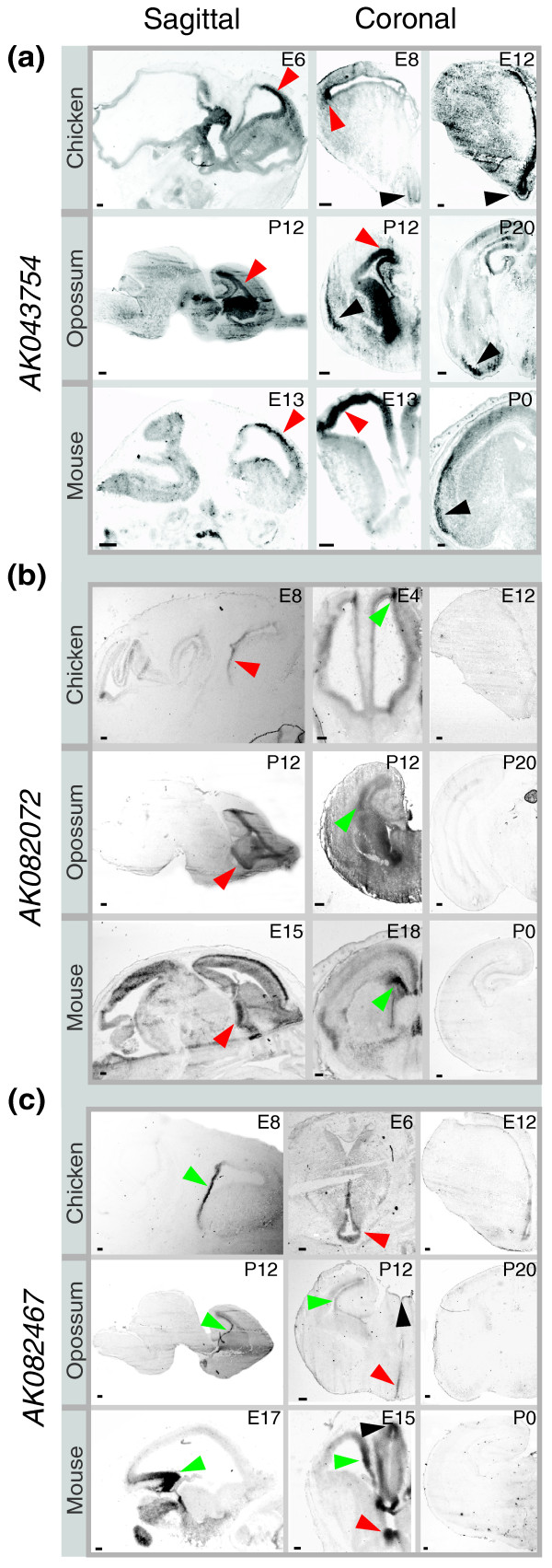
**Conservation of lncRNA expression in developing avian and mammalian brains**. **(a-c) **Digoxigenin-labeled riboprobes complementary to lncRNAs *AK043754 *(a), *AK082072 *(b), and *AK082467 *(c) were hybridized to chicken (E4, 6, 8,12), opossum (P12, 20), and mouse (E13, 15, 17, 18 and P0) brain sections. (a) *AK043754*: strong hybridization seen in the germinal zone of the telencephalic cortex at early developmental time points (red arrowheads) and then concentrated within the piriform (olfactory) cortex at later stages (black arrowheads). (b) *AK082072*: hybridization signals seen in the stria terminalis (red arrowheads) and the telencephalic ventricular zone (green arrowheads). Signal was undetectable at later developmental stages. (c) *AK082467*: hybridization signals seen in the ventricular zone of the hippocampal formation (green arrowheads), the preoptic area of the hypothalamus (red arrowheads), and the epithalamus (black arrowheads). Signal was undetectable at later developmental stages. Scale bars = 200 μm.

Probes specific to chicken, opossum, and mouse *AK043754 *orthologs hybridize to the germinal zone of the telencephalic cortex in coronal and sagittal sections of early developmental brain in all three species (red arrowheads in Figure 6a). While the neuroanatomical homology relationships between mammalian and avian brains remain controversial (see [46] for a review), most researchers agree that the telencephalic germinal zone is a source of neural progenitors in both mammals and birds [48]. We found that *AK043754*-expressing cells appear to migrate radially away from the ventricular germinal zone to the pial surface as development progresses in all three species. At later developmental stages (E12, P20, and P0 in chicken, opossum, and mouse, respectively), *AK043754 *is expressed within the piriform (olfactory) cortex (black arrowheads in Figure 6a). This conserved expression pattern - from the telencephalic germinal zone to a specific cortical substructure - implies negative selection acting on as yet unidentified *AK043754 *regulatory elements.

Early in development, chicken, opossum, and mouse prominently express *AK082072 *within the stria terminalis, a fiber bundle connecting the amygdala to the hypothalamus and other basal telencephalic regions, and the telencephalic ventricular zone (red and green arrowheads in Figure 6b). This expression is reduced at later developmental stages in all three species, suggesting that the locus has retained temporal in addition to spatial regulatory elements during amniote evolution.

The clearest example of a conserved expression pattern among chicken, opossum, and mouse is seen for *AK082467*, which hybridizes specifically to the ventricular zone of the hippocampal formation (green arrowheads in sagittal brain sections in Figure 6c), an area rich in Wnt signaling among vertebrates [49]. We also found modest conservation in expression within the preoptic area of the hypothalamus among birds and mammals and within the thalamus among mammals.

## Discussion

The application of new DNA sequencing technologies over the past decade has revealed that the vertebrate transcriptome is extensive, complex, and developmentally dynamic [5]. Most components of this interleaved network of transcripts appear to have little protein-coding capacity, and their general contribution to phenotype has often been questioned. In light of the evolving definition of a 'gene' [50,51], we argue that the lncRNA transcriptional products we characterized here exhibit signatures of evolutionary constraint on sequence and transcriptional regulation that are similar to, although less pronounced than, those for protein-coding genes. These lncRNA loci thus are biologically relevant, and should be considered genes.

### Conservation of lncRNA sequence

Reinforcing previous observations [6,16,18], our analyses of vertebrate phastCons scores across lncRNA transcriptional units revealed substantial evidence for more stringent purifying selection within proximal promoter sequences than within the transcripts themselves. Exemplifying this trend, the inferred promoter regions of *AK082072 *and *AK082467 *are highly conserved across vertebrates, with only punctuated conservation across the primary transcript sequences. Nevertheless, and in contrast to coding sequence, exonic conservation was observed to be <30% and was as low as 4% (for *Rmst*) between confirmed chicken and mouse orthologs.

Multi-exonic lncRNA loci were found to exhibit greater evolutionary constraint within exons than within introns (Figure 1a). This observation is consistent with the functionality of RNA molecules transcribed from such loci rather than, for example, functionality being imparted by the act of transcriptional elongation and chromatin remodeling. It is notable that constraint tends to be lowest on bases furthest from exon boundaries (Figure 1a). This tendency has previously been noted for protein-coding exons, where it has been associated with reduced rates of nucleotide substitution within intron-proximal exonic splicing enhancers [52]. However, lower constraint within the central portions of exons may also reflect the insertion of large transposable element sequences, which are generally free of selective constraint [53] within lncRNA exons in early eutherian evolution. In this model, large insertions into exons result in functional sequence becoming closer (in terms of fractional exonic size) to intron-exon boundaries.

Mammalian and bird *AK082072*, *Rmst*, and *AK082467 *orthologs share some, but not all, splice sites, exons, and introns (Figures 3c and 4c). Multi-species genomic sequence alignments of these loci revealed 100% sequence conservation across all examined vertebrates within a subset of donor and acceptor splice sites. Consensus splice-site motifs adjacent to exon boundaries were found to be under particularly strong constraint, as we found previously [18]. This indicates that rather than the opportunistic use of incidental splice sites by the splicing machinery, the presence and location of splice sites are evolutionarily conserved and likely to be relevant to the function(s) of these lncRNA loci.

Conservation of splice-site location may also demarcate an intron containing functional modules with secondary structures (such as primary miRNAs (pri-miRNAs)). As previously reported [17], lncRNA loci are enriched in Evofold-predicted RNA secondary structures. Two miRNAs (eutherian-conserved MIR1251 and vertebrate-conserved MIR135A2) are embedded in introns of *Rmst *alternative splice variants, indicating that this lncRNA might function as a miRNA host transcript. Similarly, numerous Evofold-predicted RNA secondary structures, which could represent as yet undiscovered miRNAs, lie within the single *AK082072 *intron.

### Conservation of lncRNA transcription

The identification of transcribed *AK082072*, *Rmst*, *AK082467*, and *AK043754 *orthologs in birds and mammals provides strong evidence for their functionality over the 310 million years since these lineages last shared a common ancestor. Over this time span, however, it appears likely that considerable evolution of each lncRNA locus has occurred. TSSs, exon structures, and poly-adenylation signals are not always well-conserved (Figures 2c, 3c, and 4c). The structure of the *AK043754 *locus, for example, appears to have been altered considerably because its proximal promoter sequence in mouse is not conserved with that in chicken (Figure 2b).

We also observed similar spatio-temporal expression patterns of each lncRNA locus among distantly related vertebrates. Far from being the result of spurious transcription, the expression of these lncRNAs might instead be tightly regulated by conserved transcription factors. Indeed, *Rmst *transcript levels are significantly reduced in *Pax2*-deficient tissues [44] and *AK043754 *has recently been reported as a direct target of the homeobox transcription factor Nanog, which is critical for embryonic stem cell pluripotency [54]. Furthermore, a described mid-hindbrain enhancer element [55] lies within an intron of *AK082072 *(Figure 3b), although whether this element facilitates expression of *AK082072 *or a neighboring protein-coding gene remains unknown.

### lncRNA functions

The observed conservation in the sequence, transcription, and expression of these lncRNA loci over hundreds of millions of years of evolution indicates that these genes must confer important functions across diverse vertebrates. Because the transcription of each of these lncRNAs is largely limited to the developing nervous system in distantly related vertebrates (Table 1), the transcripts could play critical roles in neurogenesis and neuronal differentiation in specific sectors of the developing telencephalon. The underlying molecular mechanisms could, as discussed above, involve the generation of precursor short RNAs, including pri-miRNAs. Sequence-conserved and brain-expressed lncRNA loci tend to be located adjacent to protein-coding genes that are also brain-expressed and are involved in transcriptional regulation or in nervous system development [17]. Many such lncRNA loci may thus be involved in the *cis*-regulation of neighboring protein-coding transcription factor genes [17,21]. Consequently, establishing whether expression of *AK082072 *transcriptionally regulates *Mef2C *(Figure 3a), a gene implicated in autism and intellectual disability phenotypes [56,57], warrants detailed investigation.

The study of lncRNAs in cortical development and evolution reflects relatively uncharted territory. Several transcription factors are expressed at specific times and regions during telencephalic development and cerebral cortex formation [58,59]. We hypothesize that slight differences in vertebrate developmental programs established during evolution are responsible for the radial expansion, which contributed to increased lamination of the mammalian cortex and, later, to the tangential expansion of cortical surface area that ultimately produced the human cerebral cortex [46,60,61]. The differential expression of lncRNA genes in a specific spatiotemporal pattern may promote neuronal diversity [62]. It is an exciting challenge to determine whether the lncRNAs evolved to differentially modulate the expression of relevant transcription factors or to act independently during telencephalic development and evolution. Our study represents an important first step by demonstrating that lncRNAs are conserved with respect to transcription, exon structure, and brain tissue-specific developmental expression during embryonic and early postnatal stages.

## Conclusions

Initially selected for their extensive overlap with phastCons-predicted conserved elements and mouse brain-specific expression, the three murine lncRNA loci we examined in this study exhibit several indicators of transcript functionality. Despite a lack of extensive primary sequence conservation across amniotes, we successfully identified *AK043754*, *AK082072*, *AK082467*, and *Rmst *lncRNA orthologs with modest evolutionary constraint of exon-structure and spatio-temporal transcriptional regulation in distantly related amniotes spanning at least 310 million years of evolutionary divergence. The regulatory control of transcription and splicing patterns, evolutionary conservation of exon structure, stability of mature transcripts, and presence of predicted secondary structures suggest that the transcriptional products from each locus are functional, and should therefore be considered genes. Furthermore, similarities of spatiotemporal expression patterns for these transcripts in therian and avian developing nervous systems suggest that these lncRNA loci might contribute to neurogenesis and/or neuronal differentiation programs. Experimental inquiry of these lncRNAs will hopefully elucidate their roles in vertebrate brain development and evolution.

## Materials and methods

### Multi-species sequence alignments

Regions orthologous to *AK043754*, *AK082467*, *Rmst*, and *AK082072 *(including 100 kb on either side) of the following whole-genome assemblies [63] were used in this study: frog (*Xenopus tropicalis*; xenTro2), chicken (*Gallus gallus*; galGal3), songbird (*Taeniopygia guttata*; taeGut1), lizard (*Anolis carolinensis*; anoCar1), platypus (*Ornithorhyncus anatinus*; ornAna1), opossum (*Monodelphis domestica*; monDom4), mouse (*Mus musculus*; Mm9) rat (*Rattus norvegicus*; Rn4), guinea pig (*Cavia porcellus*; cavPor3), marmoset (*Callithrix jacchus*; calJac1), macaque (*Macaca mulatta*; rheMac2), orang utan (*Pongo abelli*; ponAbe2), human (*Homo sapiens*; Hg18), chimpanzee (*Pan troglodytes*; panTro2), horse (*Equus caballus*; equCab1), dog (*Canis familiaris*; canFam2), and cattle (*Bos taurus*; bosTau3) (Figures 2, 3 and 4; coordinates provided in Table S1 in Additional file 2). We additionally used deep sequence from a chicken BAC [GenBank: AC192716] to fill a gap in the chicken whole-genome assembly. The liftOver program [64] was used to identify orthologous regions in all non-mouse species listed. We used TBA (Threaded Blockset Aligner) to generate multisequence alignments as described previously [65], and then visualized each alignment with the program Gmaj (Generalized Multiple Alignments with Java) [66]. We used evolutionarily conserved regions (ECRs; defined as genomic segments at least 100 bp in size with at least 70% sequence identity between mouse and chicken) within and between the flanking protein-coding genes as anchors to facilitate the generation of multi-species sequence alignments [67]. Finally, percent sequence identity plots across all species considered in each alignment were graphed with the program SinicView (Sequence-aligning INnovative and Interactive Comparison VIEWer) [68].

### cDNA preparation, RACE and sequencing of lncRNA orthologs

Total RNA was extracted from whole brains removed from mouse (E17), chicken (E8), and opossum (P12) using RNAeasy miniprep kit (Qiagen, Hilden, Germany) and then treated with DNAse (Roche, Basel, Switzerland). Poly-A selected RACE-ready first-strand cDNA was then generated from each RNA sample (1 μg) with the GeneRacer kit, according to the manufacturer's instructions (Invitrogen, Carlsbad, CA, USA). To obtain full-length 5' and 3' ends of opossum and chicken lncRNA orthologs, RLM-RACE (RNA ligase-mediated rapid amplification of cDNA ends) was performed with the opossum or chicken cDNA as template, and GeneRacer (Invitrogen) and gene-specific primers designed near the predicted 5' and 3' ortholog ends. Nested PCR of the RACE products was performed if needed. The resulting RACE products were cloned into the PCR4-TOPO vector (Invitrogen) and the inserts were sequenced. Using sequence information obtained from 5' and 3' RACE, PCR amplification and sequencing were performed with primers spanning the remaining portion of each ortholog. All primer sequences can be found in Table S2 in Additional file 2. Finally, the overlapping sequence fragments were merged into the predicted full-length cDNA with the program SeqMan (DNAStar, Madison, WI, USA). Identified lncRNA ortholog cDNA sequences were deposited into GenBank as follows: AK043754 chicken ortholog [GenBank:GU951674], *AK043754 *opossum ortholog [GenBank:GU951677], AK082072 opossum ortholog [GenBank:GU951678], *AK082467 *chicken ortholog [GenBank:GU951675], *AK082467 *opossum ortholog [GenBank:GU951679], *Rmst *chicken ortholog [GenBank:GU951676], and *Rmst *opossum ortholog [GenBank:GU951680].

### Tissue preparation

All animal procedures were approved by the local Ethical Review Committee and performed under license from the UK Home Office (Scientific Procedures Act, 1986). Embryonic (E11, E13, E15, and E17) and postnatal (P0, P3, and adult) mice (*M. musculus*); embryonic (E4, E6, E8, and E12) chicken (*G. gallus*), and postnatal (P4, P12, and P20) opossum (*M. domestica*) were also used. Mouse embryos were obtained by caesarean section of time-mated pregnant dams sacrificed by cervical dislocation. Chicken embryos were anesthetized on ice and then extracted from their shells. Postnatal animals were anesthetized either on ice or by pentobarbital intraperitoneal injection (45 mg/kg). Following anesthesia, animals were decapitated, and the heads or brains were immediately embedded in Tissue-Tek embedding compound (Ted Pella, Redding, CA, USA), frozen on dry ice, and then stored at -80°C. For *in situ *hybridization studies, frozen sections (10 to 15 mm) were cut with a cryostat (Leica, Wetzlar, Germany) and mounted onto Superfrost Plus slides (Thermo Fisher Scientific Inc., Waltham, MA, USA).

### *In situ *hybridization

For generation of *in situ *hybridization probes, universal degenerate oligonucleotide primers were designed from the most evolutionarily conserved regions of the selected mouse lncRNA loci and then PCR was performed using chicken, opossum, or mouse cDNA as template (primer sequences listed in Table S2 in Additional file 2). PCR products were cloned into the PCR4-TOPO vector (Invitrogen) and then sequenced to confirm authenticity. Sense and antisense probes were generated from selected PCR4-TOPO clones using T7 and T3 RNA polymerases and labeled with digoxigenin (DIG; Roche). Tissue frozen sections were postfixed with 4% paraformaldehyde in phosphate-buffered saline, deproteinized with 0.1N HCl for 5 minutes, acetylated with acetic anhydride (0.25% in 0.1 M triethanolmine hydrochloride), and prehybridized at room temperature for at least 1 hour in a solution containing 50% formamide, 10 mM Tris (pH 7.6), 200 μg/ml *Escherichia coli *tRNA, 1× Denhardt's solution, 10% dextran sulfate, 600 mM NaCl, 0.25% SDS, and 1 mM EDTA. Sections were then hybridized in the same buffer containing the DIG-labeled probe overnight at 65°C. After hybridization, sections were washed to a final stringency of 30 mM NaCl/3 mM sodium citrate at 65°C and detected using anti-DIG-alkaline phosphatase (Roche), essentially as described previously [69]. Sense probe hybridizations (Additional File 1) were used as background controls when analyzing corresponding antisense probe hybridizations.

## Abbreviations

BP: base pair; DIG: digoxigenin; E: embryonic day; ECR: evolutionarily conserved region; EST: expressed sequence tag; LNCRNA: long noncoding RNA; MIRNA: microRNA; NCRNA: noncoding RNA; ORF: open reading frame; P: postnatal day; PRI-MIRNA: primary microRNA; RACE: rapid amplification of cDNA ends; *RMST*: rhabdomyosarcoma 2 associated transcript; TBA: Threaded Blockset Aligner; TSS: transcription start site.

## Authors' contributions

RAC and LG performed the bioinformatic analyses and multi-species sequence alignments; RAC, TS, and PLO contributed to the *in situ *hybridizations; RAC carried out the RACE experiments and prepared the manuscript with assistance from KED, EDG, ZM, and CPP. ZM, CPP, EDG and RAC designed and coordinated the study. All authors read and approved the final manuscript.

## Supplementary Material

Additional file 1 **Figure S1: splice-site and poly(A)-signal conservation among *AK043754*, *AK082072*, and *AK082467 *orthologs**. Figure S2: sense probe controls for *in situ *hybridization. Click here for file

Additional file 2 **Table S1: genome coordinates used in multi-species sequence alignments**. Table S2: PCR primers used for amplification of *in situ *hybridization probes and 3' and 5' lncRNA ortholog RACE. Click here for file
