# Supplementary material for: Long noncoding RNA genes: conservation of sequence and brain expression among diverse amniotes
Source: Genome Biol. 2010 Jul 12;11(7):R72. doi: 10.1186/gb-2010-11-7-r72 (PMC2926783; doi:10.1186/gb-2010-11-7-r72)
Supplement: Additional file 1 — Figure S1: splice-site and poly(A)-signal conservation among AK043754, AK082072, and AK082467 orthologs. Figure S2: sense probe controls for in situ hybridization. [file gb-2010-11-7-r72-S1.PDF]

AK043754

Species	poly(A) signal					
M. musculus	**a**	**a**	**t**	**a**	**a**	**a**
H. sapiens	.	.	.	.	.	.
B. taurus	.	.	.	.	.	.
M. domestica	.	.	.	.	.	.
G. gallus	.	.	.	.	.	.
A. carolensis	.	.	.	.	.	.
X. tropicalis	.	.	.	.	.	.

AK082072

Species	exon 1(d)									exon 2(a)				poly(A) signal					
M. musculus	a	a	g	**G**	**T**	G	A	G	T	T	**A**	**G**	g	**a**	**a**	**t**	**a**	**a**	**a**
H. sapiens	.	.	.	.	.	.	.	.	.	.	.	.	.	.	.	.	.	.	.
B. taurus	.	.	.	.	.	.	.	.	.	.	.	.	.	.	.	.	.	.	.
M. domestica	.	.	.	.	.	.	.	.	.	.	.	.	c	.	.	.	.	.	.
G. gallus	.	.	.	.	.	.	.	.	.	-	-	-	-	-	-	-	-	-	-
A. carolensis	.	.	.	.	.	.	.	.	.	-	-	-	-	-	-	-	-	-	-
X. tropicalis	.	.	.	.	.	A	.	.	A	-	-	-	-	-	-	-	-	-	-

RMST/AK082467

Species	exon 1(d)									exon 2(a)				exon 2(d)									exon 3(a)				exon 3(d)									exon 4(a)			
M. musculus	c	t	g	**G**	**T**	G	A	G	A	T	**A**	**G**	g	t	a	g	**G**	**T**	A	A	G	G	C	**A**	**G**	c	a	a	g	**G**	**T**	A	A	A	A	C	**A**	**G**	a
H. sapiens	.	.	.	.	.	.	.	.	.	.	.	.	.	.	.	.	.	.	.	.	.	A	.	.	.	.	.	.	.	.	.	.	.	.	.	.	.	.	.
B. taurus	.	.	.	.	.	.	.	.	.	.	.	.	.	c	.	.	.	.	.	.	.	A	.	.	A	.	.	.	.	.	.	.	.	.	.	.	.	.	.
M. domestica	.	.	a	.	.	A	.	.	.	C	-	T	-	.	.	.	.	.	.	.	.	A	-	-	-	-	-	-	-	-	-	-	-	-	-	.	.	.	.
G. gallus	.	.	a	.	.	.	.	.	.	G	C	C	-	.	.	.	.	.	.	.	.	A	-	-	-	-	-	-	-	-	-	-	-	-	-	.	.	.	.
A. carolensis	t	c	c	C	.	.	.	.	.	G	C	T	-	.	.	.	.	.	.	.	.	A	-	-	-	-	-	-	-	-	-	-	-	-	-	.	.	.	.
X. tropicalis	.	.	a	.	.	.	.	.	.	A	T	T	-	-	-	-	-	-	-	-	-	-	-	-	-	-	-	-	-	-	-	-	-	-	-	.	.	.	.

Species	exon 4(d)									exon 5(a)				exon 5(d)									exon 6(a)				exon 6(d)									exon 7(a)			
M. musculus	c	a	g	**G**	**T**	G	T	G	T	A	**A**	**G**	g	a	a	g	**G**	**T**	G	A	G	C	T	**A**	**G**	c	a	a	g	**G**	**T**	A	A	G	A	T	**A**	**G**	a
H. sapiens	.	.	.	.	.	.	.	.	.	.	.	.	.	.	.	.	.	.	.	.	.	T	.	.	.	.	.	.	.	.	.	.	.	.	.	C	.	.	.
B. taurus	.	.	.	.	.	.	.	.	.	.	.	.	.	.	.	.	.	.	.	.	.	T	.	.	.	.	.	.	.	.	.	.	.	.	C	C	.	.	.
M. domestica	.	.	.	.	.	A	.	.	.	-	-	-	-	c	.	.	.	.	A	.	.	T	-	-	-	-	.	.	.	.	.	.	.	A	G	-	-	-	-
G. gallus	.	.	.	.	.	.	.	.	.	-	-	-	-	-	-	-	-	-	-	-	-	-	-	-	-	-	-	-	-	-	-	-	-	-	-	.	.	.	.
A. carolensis	.	.	.	.	.	.	.	.	.	-	-	-	-	-	-	-	-	-	-	-	-	-	-	-	-	-	-	-	-	-	-	-	-	-	-	-	-	-	-
X. tropicalis	.	.	.	.	.	A	.	.	.	-	-	-	-	-	-	-	-	-	-	-	-	-	-	-	-	-	-	-	-	-	-	-	-	-	-	.	.	.	.

Species	exon 7(d)									exon 8(a)				exon 7(d)									exon 8(a)				exon 9(d)									exon 10(a)			
M. musculus	g	a	g	**G**	**T**	A	A	G	A	T	**A**	**G**	g	t	c	a	**G**	**T**	A	T	G	T	C	**A**	**G**	g	a	a	g	**G**	**T**	A	A	G	A	G	**A**	**G**	c
H. sapiens	a	.	.	.	.	.	.	.	.	.	A	T	a	.	g	.	.	G	.	.	A	C	.	.	.	.	.	.	.	.	.	.	.	.	C	C	.	.	t
B. taurus	.	.	.	.	.	.	.	.	G	.	A	T	a	.	g	.	A	G	.	.	T	C	.	.	.	.	g	.	.	.	.	.	.	.	C	C	.	.	.
M. domestica	-	-	-	-	-	-	-	-	-	-	-	-	-	-	-	-	-	-	-	-	-	-	.	.	.	.	-	-	-	-	-	-	-	-	-	C	.	.	t
G. gallus	.	.	.	.	.	G	.	.	.	-	-	-	-	-	-	-	-	-	-	-	-	-	-	-	-	-	-	-	-	-	-	-	-	-	-	C	.	.	t
A. carolensis	-	-	-	-	-	-	-	-	-	-	-	-	-	-	-	-	-	-	-	-	-	-	-	-	-	-	-	-	-	-	-	-	-	-	-	T	.	.	t
X. tropicalis	.	.	.	.	.	.	.	.	T	-	-	-	-	-	-	-	-	-	-	-	-	-	-	-	-	-	-	-	-	-	-	-	-	-	-	C	.	.	.

Species	exon 10(d)									exon 11(a)				exon 11(d)									exon 12(a)			
M. musculus	a	a	g	**G**	**T**	A	T	G	T	C	**A**	**G**	g	c	a	g	**G**	**T**	A	C	A	C	C	**A**	**G**	g
H. sapiens	.	.	.	.	.	.	.	.	.	T	.	.	.	.	.	.	.	.	.	.	.	.	-	-	-	-
B. taurus	.	.	.	.	.	.	G	.	.	T	.	.	.	.	.	.	.	.	.	.	.	.	-	-	-	-
M. domestica	.	.	.	.	.	.	.	.	.	G	.	.	.	a	.	.	.	.	.	T	.	T	-	-	-	-
G. gallus	.	.	.	.	.	.	.	.	.	-	-	-	-	-	-	-	-	-	-	-	-	-	-	-	-	-
A. carolensis	.	t	.	.	.	.	G	.	.	-	-	-	-	-	-	-	-	-	-	-	-	-	-	-	-	-
X. tropicalis	.	.	.	.	.	.	G	.	.	-	-	-	-	-	-	-	-	-	-	-	-	-	-	-	-	-

SUPPLEMENTARY FIGURE 1. Splice-site and poly(A)-signal conservation among AK043754, AK082072, and AK082467 orthologs. Selected views of TBA alignment blocks for the three lncRNAs are shown, highlighting common canonical splice sites and poly(A) signals (dark green), conserved flanking sequence (light green), and substitutions (yellow). Uppercase and lowercase letter reflect nucleotides in annotated mouse lncRNA introns and exons, respectively.

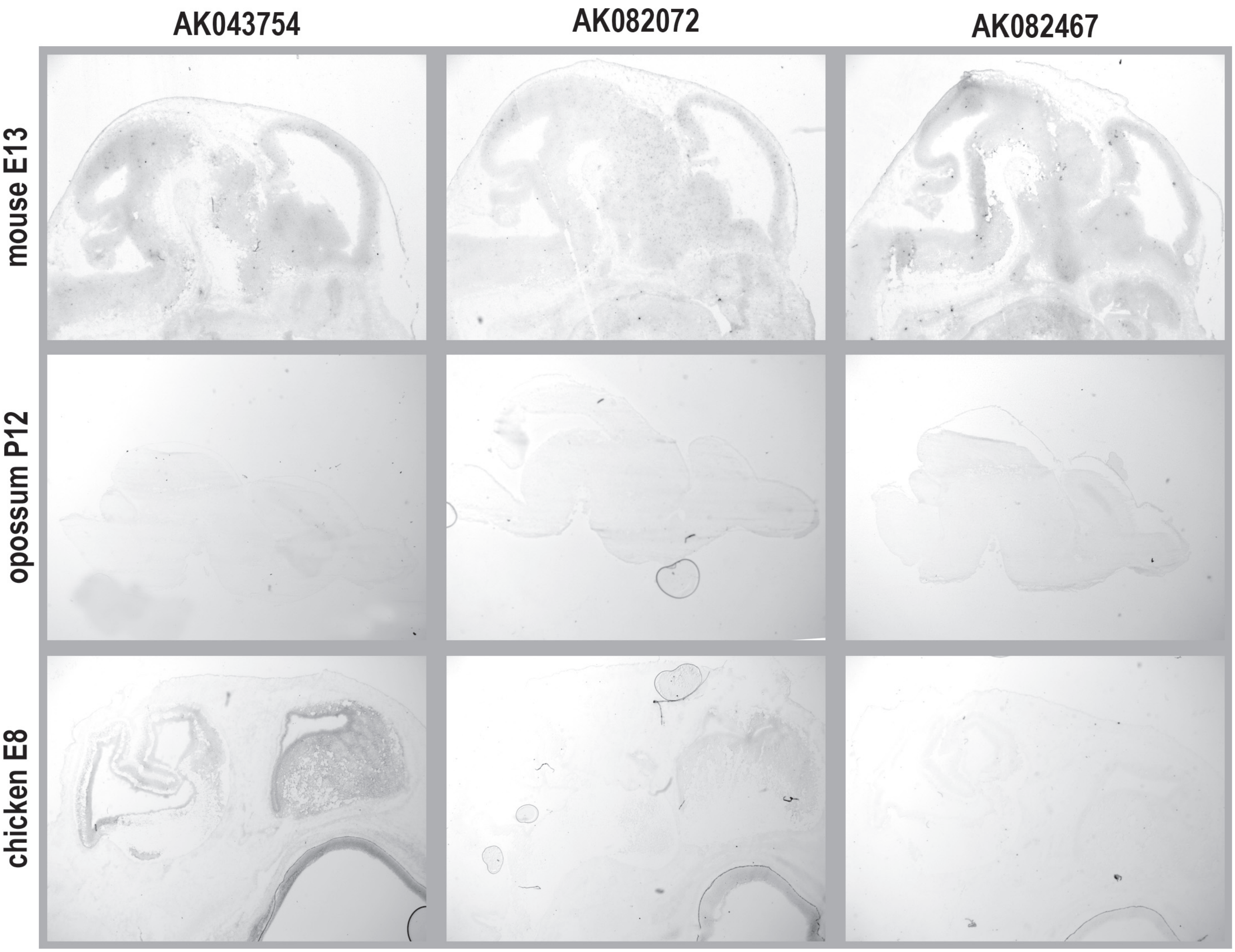

SUPPLEMENTARY FIGURE 2. Sense probe background controls for *in situ* hybridization. Digoxigenin-labelled riboprobes transcribed in the same direction as *AK043754, AK082072,* and *AK082467* were hybridized to sagittal sections of mouse (E13), opossum (P12), and chicken (E8) brains in order to quantify the amount of background hybridization associated with each sequence. These images were used as sense controls when analysing images in **Figures 5-6**.
