# Supplementary material for: Long noncoding RNA genes: conservation of sequence and brain expression among diverse amniotes
Source: Genome Biol. 2010 Jul 12;11(7):R72. doi: 10.1186/gb-2010-11-7-r72 (PMC2926783; doi:10.1186/gb-2010-11-7-r72)
Supplement: Additional file 2 — Table S1: genome coordinates used in multi-species sequence alignments. Table S2: PCR primers used for amplification of in situ hybridization probes and 3' and 5' lncRNA ortholog RACE. [file gb-2010-11-7-r72-S2.PDF]

SUPPLEMENTARY TABLE 1. Genome coordinates used in multi-species sequence alignments.

AK043754	
Anolis carolinensis	scaffold_58:590628-829740
Bos taurus	chrUn.003.143:277972-425644
Bos taurus	chr5:91556797-91853326
Callithrix jacchus	Contig7336:5198-130368
Callithrix jacchus	Contig8225:1004-83231
Callithrix jacchus	Contig5278:2641-177019
Callithrix jacchus	Contig1369:409419-473544
Canis familiaris	chr27:35728746-36099775
Cavia porcellus	scaffold_28:7416736-7792077
Equus caballus	chr6:41254868-41610450
Gallus gallus	chr1:50271934-50460406
Homo sapiens	chr12:13381429-13791551
Macaca mulatta	chr11:13703033-14114271
Monodelphis domestica	chr8:106039041-106678870
Mus musculus	chr6:135,563,251-135,764,980
Ornithorhynchus anatinus	Contig21725:3636-15415
Ornithorhynchus anatinus	chr2:30880789-30889071
Ornithorhynchus anatinus	Contig3513:1070-37416
Pan troglodytes	chr12:13753246-14167631
Pongo abelii	chr12:13760066-14181822
Rattus norviegus	chr4:172484680-172906812
Taeniopygia guttata	chr1A:47793773-47979659
Xenopus tropicalis	scaffold_19:1479362-1536038
AK082072	
Anolis carolinensis	scaffold_184:1120998-1313616
Bos taurus	chr7:79533137-79880172
Callithrix jacchus	Contig5:1817246-1931334
Callithrix jacchus	Contig2424:3807-239138
Canis familiaris	chr3:22948477-23256091
Cavia porcellus	scaffold_1:16394909-16745650
Equus caballus	chr14:78202142-78493067
Gallus gallus	chrZ:59316385-59426058
Homo sapiens	chr5:87555757-87914940
Macaca mulatta	chr6:84505587-84872043
Monodelphis domestica	chr3:215805845-216276949
Mus musculus	chr13:84,064,908-84,304,377
Ornithorhynchus anatinus	chr1:6655271-6905052
Ornithorhynchus anatinus	Contig3118:10349-26603
Pan troglodytes	chr5:27068971-27433822
Pongo abelii	chr5:88759990-89125222
Rattus norviegus	chr2:13493134-13570930
Taeniopygia guttata	chrZ:12037011-12069084
Xenopus tropicalis	scaffold_76:2413961-2548898
AK082467/RMST	
Anolis carolinensis	scaffold_58:3429997-3719782
Bos taurus	chr5:59961661-60479913
Callithrix jacchus	Contig4279:493-142733
Callithrix jacchus	Contig1650:32836-400728
Canis familiaris	chr15:39888313-40359167
Cavia porcellus	scaffold_9:4326673-4796732
Equus caballus	chr28:21845027-22294927
Gallus gallus	chr1:48039282-48262518
Homo sapiens	chr12:96165244-96723184
Macaca mulatta	chr11:98474751-98994694
Monodelphis domestica	chr8:70392459-71105419
Mus musculus	chr10:91,444,491-91,727,923
Ornithorhynchus anatinus	Ultra443:6716447-7010969
Ornithorhynchus anatinus	chr2:51132131-51161181
Pan troglodytes	chr12:98260913-98837145
Pongo abelii	chr12:98468956-99042606
Rattus norviegus	chr7:28872383-29380125
Taeniopygia guttata	chr1A:45502853-45716086
Xenopus tropicalis	scaffold_5:305355-521924

SUPPLEMENTARY TABLE 2. List of PCR primers used for amplification of *in situ* hybridization probes and 3’ and 5’ lncRNA ortholog RACE

**Primers for mouse *in situ* probes (FIGURE 5)**			
lncRNA	species	forward	reverse
AK043754	mouse	ccttcctgatggtcctctca	ctcccccacttgacacagat
AK082072	mouse	tgctgattaaggttgcttgg	ctagaattggcatggcatca
AK082467	mouse	gcaggcagcactaacactca	caggcacaaagattccgaac

**Primers for amniote *in situ* probes (FIGURE 6) and lncRNA ortholog amplification (FIGURES 2-4)**			
lncRNA	species	forward	reverse
AK043754	mouse, chicken	cagggatcmaytgagattctg	cctatktacarsttttrtacatcacaaa
AK043754	opossum	tgtaaataagtatttctcattttgga	tttgtgatgtataaaagttgtaaatagg
AK082072	chicken	gctcccattggagaacagaa	cgaaccgataaacgctgtct
AK082072	opossum	caaggtattgaaatgctcacct	ctttattgacaatctctgttctgaaagt
AK082072	mouse	tgctgattaaggttgcttgg	ctagaattggcatggcatca
AK082467	mouse, opossum, chicken	atgggtkcagsgtgatct	tgttccataccattaactcmgaaca
RMST	opossum	gcacggactgctgggcag	cgattaaacttctccgatgtggttgc
RMST	chicken	gcacgggctgctgggcag	cgattaaacttctcagacatggtagcaact

**Primers for lncRNA ortholog RACE (FIGURES 2-4)**			
lncRNA	species	RACE primer	nested primer
AK043754 5'	opossum	ctcccctcccccgcccctgctt	catccctcctataacaaacaactttcaaag
AK043754 3'	opossum	gggggtgggggtttagggtggaggg	not needed
AK043754 5'	chicken	aagtgtcttctctgcccccctccagaatctca	gttgatccctgcttgtccaaagtcagaaagagt
AK043754 3'	chicken	tggacaagcagggatcaac	cagttcagcattgtttaaaggatgtggaggg
AK082072 5'	opossum	gctcattaaattttctgttctccaatgggagg	gaactgcatgcttttgcttcgaatgatc
AK082072 3'	opossum	gacacagcattagccctggctgctgtt	gaggccagtgcagagttcaggattggtc
AK082467/RMST 5'	opossum	tagatcaccctgcacccatgacaaagggc	catccctcctataacaaacaactttcaaag
AK082467 3'	opossum	ctatggccggtctgaccccaatccata	atggtgctatgcaattcatcaattcttac
AK082467/RMST 5'	chicken	agattagatcacgctgcacccatcacaat	ctgcacccatcacaatagacttcacagtgaa
AK082467 3'	chicken	gctatggccaatctgacctcaatctatgc	atggcggtgtacaattcatcagttcttac
